# Impact of a Real-Time Feedback Device on the Quality of Chest Compressions Performed by Laypersons: A Randomised Controlled Trial

**DOI:** 10.3390/jcm15124787

**Published:** 2026-06-19

**Authors:** Louise D’Argent, Eline Vandenabeele, Olivier Hoogmartens, Didier Desruelles, Nathalie Charlier, Marc Sabbe

**Affiliations:** 1 Department of Emergency Medicine, University Hospitals Leuven, 3000 Leuven, Belgium; louise.dargent@student.kuleuven.be (L.D.); eline.vandenabeele@student.kuleuven.be (E.V.); marc.sabbe@uzleuven.be (M.S.); 2 Department of Public Health and Primary Care, The Institute of Healthcare Policy, KU Leuven, 3000 Leuven, Belgium; 3 Research Unit Emergency Medicine, Department of Public Health and Primary Care, KU Leuven, 3000 Leuven, Belgium; 4 Department of Pharmaceutical and Pharmacological Sciences, KU Leuven, 3000 Leuven, Belgium

**Keywords:** cardiopulmonary resuscitation (CPR), real-time feedback device, basic life support (BLS), randomised controlled trial, chest compression parameters, skill retention

## Abstract

**Background/Objectives:** This prospective, randomised controlled trial aimed to evaluate whether using a real-time feedback device during basic life support (BLS) training for laypersons improves chest compression quality immediately after training and at the four-month follow-up. **Methods:** Participants were randomly assigned to a control group (standard BLS training) or an intervention group (BLS training with a real-time feedback device). All participants completed a standardised 2-h BLS course, followed by a 4-min practical assessment immediately after training and at the four-month follow-up. The primary outcomes were chest compression rate and depth, while the secondary outcomes were correct hand position, full chest recoil and flow fraction. These compression parameters were compared within and between groups at both time points. **Results:** Data from 101 participants were analysed. Both groups showed significantly decreased mean and adequate compression rates over time, but only the intervention group demonstrated significantly better performance at follow-up. The mean compression depth was approximately 5 cm in both groups; however, the proportion of adequate compression depth was low and did not differ significantly within or between groups. Correct hand position was consistently higher in the intervention group across both assessments. Full chest recoil improved in both groups, whereas flow fraction increased only in the control group. **Conclusions**: Incorporating real-time feedback devices into layperson BLS training leads to superior performance in selected chest compression parameters, particularly compression rate and hand position. Therefore, real-time feedback devices can be a valuable adjunct to standard BLS training to enhance skill retention over time.

## 1. Introduction

Out-of-hospital cardiac arrest (OHCA) has a worldwide incidence of approximately 3.8 million cases annually and a survival rate of only 10%, making it one of the leading causes of death globally [1,2,3]. Each link in the ‘Chain of Survival’—consisting of early recognition and call for help, early bystander cardiopulmonary resuscitation (CPR), early defibrillation and standardised post-resuscitation care—must be optimised to maximise both survival and neurological outcomes following cardiac arrest [4].

Given that prompt CPR initiation during cardiac arrest can double or even triple survival, bystander CPR has become a critical and potentially life-saving intervention. According to the European Resuscitation Council (ERC) guidelines, high-quality chest compressions are essential for successful resuscitation. Accurate hand placement on the lower half of the sternum, a compression depth of 5–6 cm, a rate of 100–120 compressions per minute, full chest recoil after each compression and minimal interruptions in chest compressions characterise high-quality chest compressions [5]. However, chest compression quality is frequently suboptimal and tends to deteriorate during ongoing resuscitation [6,7,8]. Furthermore, a decline in CPR skills as early as three to six months after initial training has been widely observed [9,10].

Several real-time feedback devices have already been developed to enhance CPR performance and are recommended by the ERC for use during CPR training [11,12]. Real-time feedback devices may offer particular advantages in community-based CPR training programmes because they provide immediate objective feedback without requiring continuous instructor correction. Such devices could help standardise training quality, reinforce correct psychomotor skills and potentially improve retention of CPR competencies among lay rescuers. The impact of these devices on chest compression quality by healthcare professionals has been extensively evaluated. Although the available evidence remains heterogeneous, the use of real-time feedback devices during CPR has shown promising results [7,8,13,14,15]. Nonetheless, the impact of these devices on the quality of chest compressions performed by laypersons remains largely unknown, with several studies showing inconsistent findings [11,16,17]. To our knowledge, no studies have evaluated the effect of real-time feedback devices on the quality of chest compressions performed by adult laypersons at predefined time points after standard basic life support (BLS) training. Although several studies have demonstrated improvements in CPR performance when real-time feedback devices are used during training, most investigations have focused on immediate post-training outcomes, healthcare professionals, or mixed populations of participants. Furthermore, evidence regarding long-term skill retention among adult laypersons remains limited and inconsistent. As bystander CPR is often performed months or years after initial training, understanding whether feedback-assisted training improves retention of CPR skills is of particular clinical relevance.

This study aimed to evaluate whether incorporating a real-time feedback device into standard BLS training enhances the acquisition and retention of high-quality chest compression skills among adult laypersons. By improving CPR skill retention, feedback-assisted training may contribute to better bystander CPR quality and ultimately strengthen the early links in the Chain of Survival.

## 2. Materials and Methods

### 2.1. Study Design

This prospective, randomised controlled trial assessed whether incorporating a real-time feedback device into BLS training for laypersons improves chest compression quality immediately after training and at a 4-month follow-up. This study obtained approval from the Ethics Committee Research UZ/KU Leuven (approval number MP031263), conforming to the ethical principles outlined in the Declaration of Helsinki, the International Council for Harmonisation Good Clinical Practice (ICH-GCP) guidelines and the Belgian law of 7 May 2004 on experiments involving the human person. All participants provided written informed consent before participation and could withdraw at any time without consequences.

### 2.2. Study Setting and Population

This study was conducted in two companies located in two cities (Leuven and Kortrijk) in Belgium and participants were individuals representative of the general population. A total of 174 participants were anticipated according to a power calculation assuming a 10% decline in CPR performance over a three- to six-month period, with a significance level of α < 0.05. Inclusion criteria were participants aged 18 years or older. Exclusion criteria were previous BLS or advanced life support (ALS) training within the past five years, or physical disabilities preventing BLS performance.

### 2.3. Study Protocol

Participants were randomly assigned to a control group (BLS training only) or an intervention group (BLS training with a real-time feedback device). Both groups attended two sessions. Randomisation was based on participants’ registration for the first session. The lead researchers randomly allocated time slots for the first session to the control and intervention groups, with an allocation ratio of 1:2, using a computer-generated random number list. Subsequently, participants were invited to anonymously register for a time slot for the first training session and were then assigned to their respective groups by research assistants. Pseudonymisation was achieved by drawing a sealed envelope containing a unique study number for each participant.

In the first session, participants underwent a standardised 2-h BLS training in groups of 7–15, led by a certified BLS instructor, followed immediately by a practical BLS test assessing chest compression parameters. At the 4-month follow-up, a second practical BLS test was conducted evaluating retention of chest compression quality over time. The practical test during both sessions comprised a 4-min BLS test using a 30:2 compression-to-ventilation ratio. Both BLS training and practical tests were performed with or without a real-life feedback device, depending on group allocation. Apart from the real-time feedback provided by the device, participants did not receive any additional feedback regarding the quality of their practical test performance.

### 2.4. Data Collection

At the start of BLS training, participants provided demographic information, including sex and age. Baseline characteristics were compared between groups to identify potential differences that could influence the results. Additionally, participants were asked about previous BLS or ALS training or real-life BLS experience.

Chest compression quality was assessed using the following parameters: total number of compressions during the test; mean compression rate (mean number of compressions per minute) (min^−1^); adequate compression rate (percentage of compressions within the optimal 100–120 min^−1^ range) (%); mean compression depth (cm); adequate compression depth (percentage of compressions within the optimal 5–6 cm range) (%); correct hand position (%); full chest recoil (percentage of compressions fully released) (%); and flow fraction (percentage of compression time without pauses ≥ 3 s) (%). Primary outcomes were mean and adequate compression rate and depth. Secondary outcomes were correct hand position, full chest recoil and flow fraction. Correct hand position was defined according to the ERC guidelines as placement on the lower half of the sternum. This was objectively measured by the manikin’s internal sensors, which log a compression as incorrect if the pressure point deviates from this anatomically designated zone.

This study used the real-time feedback device CPRcard (Laerdal Medical, Inc., Stavanger, Norway). Both the BLS training session and practical tests were conducted on Laerdal Resusci Anne QCPR torso manikins (Laerdal China Ltd., Hangzhou, China). Chest compression performance metrics obtained during the practical tests were recorded and stored on SimPad (Laerdal Medical, Inc., Norway).

### 2.5. Statistical Analysis

The recorded chest compression parameters stored on SimPad were downloaded for offline analysis. All data were entered in pseudonymised form into a database (Microsoft Excel 365, Microsoft Corporation, Redmond, WA, USA) and analysed using SPSS version 30 (SPSS Inc., Armonk, NY, USA).

Demographic differences between the control and intervention groups were evaluated using Pearson’s chi-square test. Normality of data distribution was assessed via histogram analysis. Chest compression outcomes are presented as mean ± standard deviation (SD). Paired *t*-tests were used for within-group comparisons of chest compression parameters between sessions 1 and 2, and two-sided independent *t*-tests were used for between-group differences. A *p* value < 0.05 was considered statistically significant.

To evaluate the longer-term retention of BLS skills, we calculated the relative rate of skill decay for all performance parameters. This study defined skill decay as the percentage change in mean performance between the initial assessment (session 1) and the follow-up assessment (session 2), calculated using the formula: [(M_Session 2 − M_Session 1)/M_Session 1] × 100.

Furthermore, to assess the clinical significance of the observed differences between the two groups, we determined effect sizes using Cohen’s d. We utilised a pooled SD for between-group comparisons. Following established statistical conventions, d values of 0.2, 0.5 and 0.8 indicate small, medium and large effects, respectively.

Data analysis followed a complete-case approach; participants with missing follow-up data or those meeting exclusion criteria were excluded from the final analysis, and no intention-to-treat analysis was performed. Normality was visually confirmed via histogram analysis.

### 2.6. AI Statement

During the preparation of this work the authors used DeepL Translate version 26.37 (DeepL SE) to confirm using proper academic English language. After using this tool, the authors reviewed and edited the content as needed and take full responsibility for the content of the published article.

## 3. Results

This study recruited 226 participants through convenience sampling. At the start of the first session, a pilot study encompassing 19 participants was undertaken to evaluate the study’s feasibility and to train the BLS instructor in the standardised protocol. These 19 pilot participants were excluded from the final analysis.

**Figure 1:**
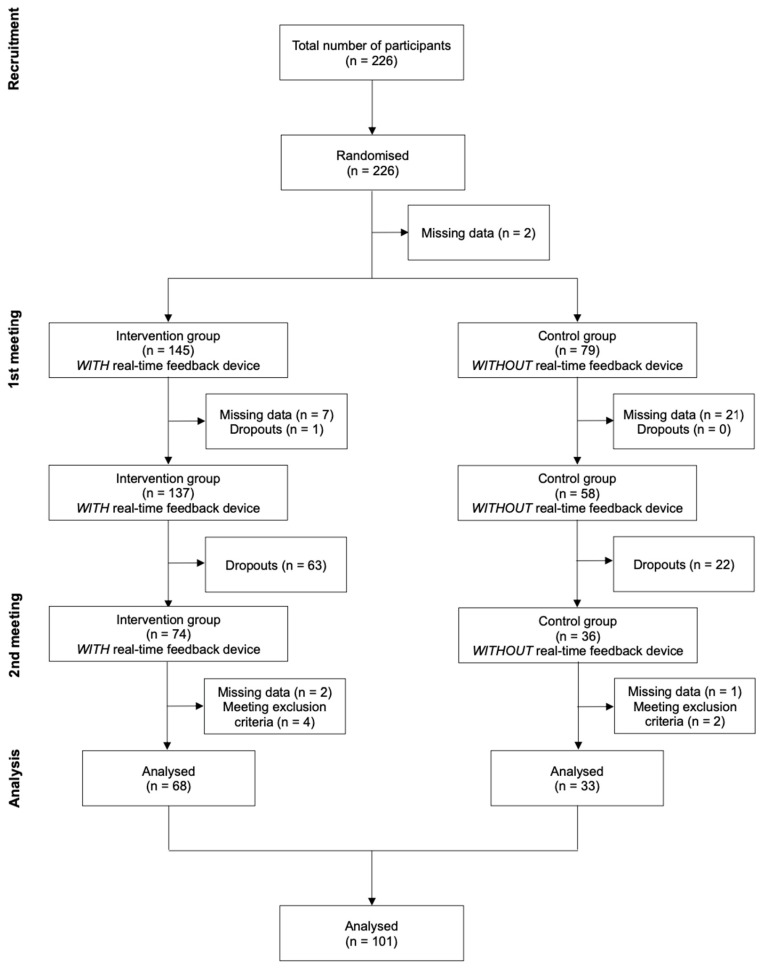
CONSORT flow diagram.

Following the two sessions and after accounting for missing data and participant dropouts caused by no-show, six participants were further excluded based on the predefined exclusion criteria. Ultimately, 33 participants remained in the control group and 68 in the intervention group (Figure 1). The unequal group sizes resulted primarily from differential attrition between sessions, particularly within the control group.

As shown in Table 1, no demographic characteristics significantly differed between the two groups. Histogram analyses showed no significant skewness or outliers, indicating that the data follow a normal distribution suitable for further analysis.

Table 2 and Table 3 present the within-group differences in chest compression parameters between sessions 1 and 2. The mean compression rate decreased from 108.48 min^−1^ in session 1 to 94.03 min^−1^ in session 2 (*p* < 0.001) in the control group and from 113.07 min^−1^ to 102.87 min^−1^ (*p* < 0.001) in the intervention group. Regarding between-group comparisons, the mean compression rate did not significantly differ between the two groups in session 1 (*p* = 0.060) (Table 4) but showed a significant difference in session 2, with the intervention group achieving a compression rate within the recommended 100–120 min^−1^ range, whereas the control group did not (*p* = 0.008) (Table 5).

The adequate compression rate decreased significantly in each group (*p* < 0.001), from 66.88% to 35.52% in the control group (Table 2) and from 72.88% to 53.68% in the intervention group (Table 3). It also significantly differed between both groups in session 2 (*p* = 0.028), with the intervention group demonstrating better performance (Table 5).

The mean compression depth in the control group decreased from 5.06 cm in session 1 to 4.86 cm in session 2, the latter falling below the recommended 5–6 cm range (Table 2). In the intervention group, it decreased slightly from 5.18 cm to 5.06 cm (Table 3). Nonetheless, both the within-group and between-group differences were not significant (Table 2, Table 3, Table 4 and Table 5).

The adequate compression depth remained low across both sessions for both groups. In the within-group comparison, only the intervention group showed a significant decline from 54.62% to 47.26% (*p* = 0.040) (Table 3). Meanwhile, no significant between-group differences were found (Table 4 and Table 5).

Correct hand position was significantly higher in the intervention group than in the control group in both session 1 (93.31% vs. 76.09%) and session 2 (88.35% vs. 73.64%) (*p* = 0.011 and 0.045, respectively) (Table 4 and Table 5).

The proportion of full chest recoil improved in both groups in session 2, increasing from 65.52% to 78.36% in the control group (*p* = 0.044) and from 54.50% to 69.91% in the intervention group (*p* < 0.001) (Table 2 and Table 3). However, no significant between-group differences were observed (Table 4 and Table 5).

Flow fraction increased in the control group, from 64.88% in session 1 to 68.12% in session 2 (*p* = 0.046) (Table 2). Conversely, no significant difference was observed in the intervention group (Table 3). Between-group differences were not significant in either session (Table 4 and Table 5).

**Figure 2:**
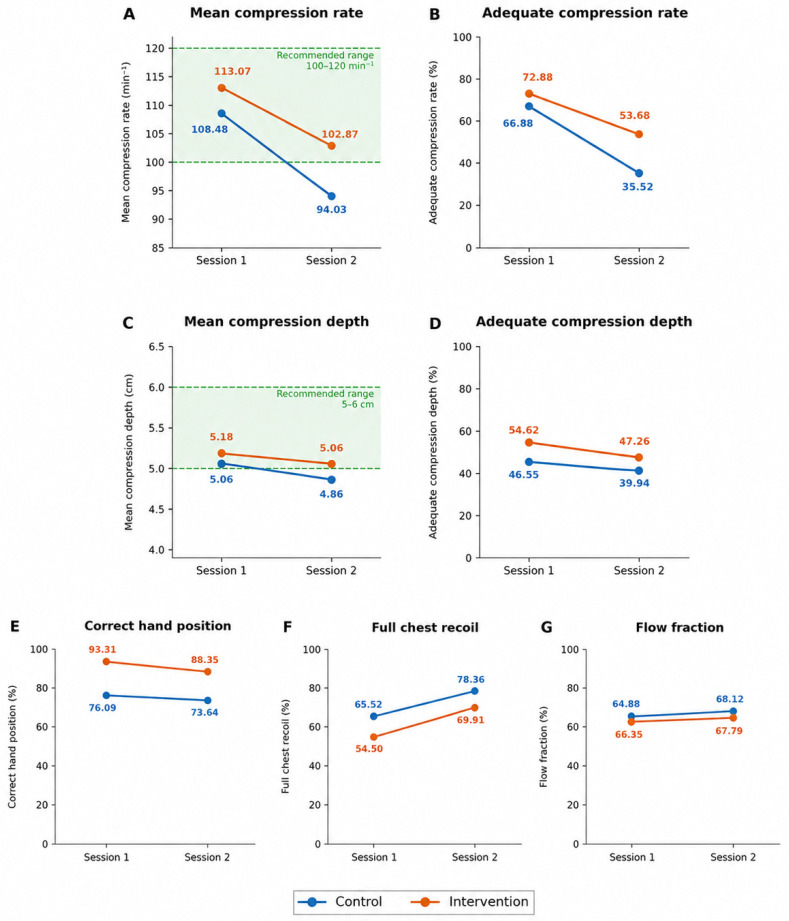
Changes in chest compression performance parameters between session 1 and session 2 in the control and intervention groups. (**A**) Mean compression rate; (**B**) Adequate compression rate; (**C**) Mean compression depth; (**D**) Adequate compression depth. (**E**) Correct hand position; (**F**) Full chest recoil; (**G**) Flow fraction.

To assess the clinical significance of the differences between groups at the 4-month follow-up, effect sizes (Cohen’s d) were calculated for all primary and secondary outcomes (Table 5). The use of a real-time feedback device during training was associated with a moderate effect on mean compression rate maintenance (d = 0.58). Although both groups showed decline in performance over time, the intervention group adhered to guideline-recommended pacing more significantly than the control group (*p* = 0.008). Moderate effects were observed for adequate compression rate (d = 0.47) and total number of compressions (d = 0.55). Conversely, the feedback device had minimal impact on compression depth. Between-group differences for mean compression depth (d = 0.23) and adequate compression depth (d = 0.25) were small, consistent with the lack of statistical significance observed for these parameters (*p* = 0.267 and *p* = 0.248, respectively). Among secondary outcomes, correct hand position remained higher in the intervention group, with a moderate effect size (d = 0.46), than in the control group (*p* = 0.045), whereas effects on full chest recoil and flow fraction were negligible at follow-up. Figure 2 represents an overview of the changes in chest compression performance parameters between session 1 and session 2 in the control and intervention groups.

To quantify skill decay over the four-month follow-up period, we calculated the percentage change in performance between sessions 1 and 2 (Table 6). The adequate compression rate declined in both groups; however, the magnitude of decay was markedly more pronounced in the control group (−46.9% vs. −26.3%). Therefore, the use of a real-time feedback device significantly reduced temporal accuracy loss in chest compressions. Additionally, the mean compression rate was more stable in the intervention group, with a decay of only 9.0%, compared with 13.3% in the control group (Table 6). Importantly, the mean compression rate was maintained within the recommended clinical target range of 100–120 min^−1^ at follow-up (102.87 min^−1^) in the intervention group but declined to a suboptimal level (94.03 min^−1^) in the control group. Conversely, the decline in adequate compression depth was comparable between both groups (control: −14.2%; intervention: −13.5%); thus, skill decay for depth may be less affected by initial feedback-assisted training than temporal parameters. Correct hand position showed minimal skill decay in both groups (control: −3.2%; intervention: −5.3%), with the intervention group consistently maintaining a higher overall level of accuracy. Interestingly, no skill decay was observed for full chest recoil and flow fraction. Instead, both groups showed improvements at 4-month follow-up. Full chest recoil increased by 19.6% in the control group and 28.3% in the intervention group, while flow fraction rose slightly by 5% and 2.2%, respectively.

## 4. Discussion

This randomised controlled trial evaluated whether integrating a real-time feedback device into standard BLS training improves chest compression quality performed by laypersons, both immediately after training and at 4-month follow-up. Compared with standard training alone, feedback-assisted training led to superior compression rate performance and better adherence to guideline-recommended pacing at follow-up. Conversely, compression depth showed no clinically significant improvement. Overall CPR performance declined over time in both groups, consistent with previous reports of significant skill deterioration within 3–6 months after initial training [9,10].

Of the assessed parameters, compression rate was most strongly influenced by feedback-assisted training. Although mean compression rate decreased over time in both groups, the intervention group maintained values within the recommended range of 100–120 compressions per minute at follow-up, whereas the control group did not. Furthermore, adequate compression rate remained significantly higher in the intervention group. Therefore, real-time feedback devices may be particularly efficacious in reinforcing CPR’s temporal aspects (e.g., pacing), which are challenging for lay rescuers to self-regulate without external cues.

Compression depth was not significantly improved by the intervention, representing an important negative finding. Despite receiving continuous audiovisual feedback, the intervention group failed to achieve adequate depth in more than half of compressions by session 2, and the proportion of adequate depth declined over time in both groups—even as mean depth remained near guideline targets. This aligns with previous research identifying compression depth as one of the most challenging components of high-quality CPR for lay rescuers [18].

The absence of an effect on depth, in contrast to the clear benefit seen for compression rate, suggests that real-time feedback devices may be more effective at correcting temporal parameters than force-dependent ones. Physical characteristics such as body weight and upper-body strength, combined with fear of causing harm and rescuer fatigue, likely limit what audiovisual prompting alone can achieve. Addressing compression depth may therefore require targeted physical conditioning, extended training formats, or alternative feedback modalities beyond current device capabilities.

The intervention group demonstrated a higher degree of consistency in achieving correct hand positioning during both sessions, suggesting that real-time feedback may improve accurate hand placement. This effect can be attributed to the visual positioning cues provided by the feedback device once positioned on the chest. However, this advantage relies on the correct initial device placement; incorrect positioning may result in systematically incorrect hand placement throughout the entire resuscitation attempt, representing a potential limitation of feedback-assisted CPR.

For chest recoil or flow fraction, no significant between-group differences were observed. The training provided to the groups was equivalent because the feedback device was not designed to provide feedback on manual ventilations. Chest recoil improvement seen in both groups over time may reflect increased familiarity with CPR technique following initial training rather than a specific effect of the feedback device.

The findings have significant clinical implications in relation to the Chain of Survival. Given that OHCA continues to be associated with survival rates of only 10%, optimising bystander CPR quality remains a critical determinant of patient outcomes. Despite persistent challenges in maintaining adequate compression depth, the superior performance of the intervention group in compression rate and hand positioning suggests that real-time feedback devices can meaningfully enhance bystander CPR quality. Although improvements in compression rate and hand position are associated with higher-quality CPR and may theoretically contribute to improved patient outcomes, this study did not evaluate clinical outcomes such as return of spontaneous circulation, survival to hospital discharge or neurological recovery. Therefore, the extent to which the observed improvements translate into meaningful patient benefit remains uncertain and warrants further investigation in real-world settings.

Our study demonstrates that integrating real-time feedback devices effectively mitigates the skill decay of compression rate, which is fundamentally linked to haemodynamic stability and cardiac output. The compression rate must be maintained within the guideline-recommended range of 100–120 min^−1^ to optimise coronary perfusion pressure and ensure adequate cerebral blood flow during resuscitation [5]. The ability of the intervention group to maintain the compression rate within the critical threshold at four-month follow-up emphasises the potential of feedback devices to support longer-term skill retention and counteract the well-documented decline in CPR performance among lay rescuers. By limiting the skill decay in compression rate over time, feedback-assisted training improves the rapid loss of psychomotor skill proficiency, a fundamental limitation of conventional CPR education.

Although several landmark studies have demonstrated that real-world bystander CPR performed by laypersons significantly increases survival rates and favourable neurological outcomes after out-of-hospital cardiac arrest, direct clinical data evaluating the deployment of feedback devices by lay rescuers in actual emergencies remain scarce [19]. Investigating real-world clinical outcomes in this specific population presents profound logistical and ethical challenges, rendering direct tracking of skill performance during real-life resuscitations challenging [20]. Consequently, prospective manikin-based simulation studies—such as the one presented here—remain a widely accepted surrogate methodology for educational research aimed at objectively quantifying CPR psychomotor skill acquisition and long-term retention over time.

This study has several methodological strengths supporting the findings’ validity. The prospective randomised controlled design provides high-level evidence regarding the effectiveness of real-time feedback devices used by laypersons. Importantly, by including a four-month follow-up, medium-term skill retention could be assessed, addressing a limitation of many prior studies that focus solely on immediate post-training performance. However, longer follow-up periods are required to determine whether the observed benefits persist beyond four months. Furthermore, implementing standardised BLS training, delivered by a single certified instructor, ensured that all participants received uniform baseline instruction, thereby minimising potential educational bias. Finally, data reliability was further strengthened by the independent review of SimPad records and performance data.

However, several limitations should be acknowledged. First, CPR performance was assessed in a simulated environment using Laerdal Resusci Anne© QCPR manikins. Although these manikins are widely accepted as training standards, their chest stiffness and damping characteristics do not perfectly replicate the biofidelity of a human thorax. Consequently, the absolute values for compression depth and chest recoil obtained in this study may not fully translate to clinical practice. Secondly, participants received training in groups ranging from 7 to 15 individuals. The potential influence of group size on learning outcomes was not specifically analysed and therefore cannot be excluded as a contributing factor to performance differences. Thirdly, the practical assessment was limited to 4 min; in real-world OHCA scenarios, bystander CPR may extend beyond 4 min before emergency medical services arrive. Consequently, the findings may not fully reflect the impact of rescuer fatigue on CPR quality over a more extended duration. Finally, as previously mentioned, although randomisation was used, potential confounding factors such as participant motivation, physical fitness, upper-body strength, prior informal CPR exposure, or individual learning ability were not formally measured and may have influenced CPR performance independently of the intervention. Physical strength could be a particularly important factor since the intervention failed to significantly improve compression depth.

Several potential sources of bias should be acknowledged. The randomisation procedure was based on participants registering for pre-assigned training time slots rather than direct individual allocation, which may have introduced selection bias if certain participant characteristics influenced time-slot preferences. Blinding of participants was not feasible because the intervention required the visible use of a real-time feedback device during training and assessment, creating a risk of performance bias. Participants assigned to the intervention group may have been more motivated or engaged because they were aware of receiving additional technological support. Although chest compression outcomes were recorded automatically by the manikin software device SimPad (Laerdal Medical, Inc., Norway), investigators were aware of group allocation, and therefore a degree of detection bias cannot be excluded. In addition, the final analysed groups were unequal in size (33 versus 68 participants) owing to differential dropout during follow-up. While baseline characteristics were comparable, this imbalance may have reduced statistical power and introduced attrition bias. Missing data and participant dropouts were handled by analysing only participants with complete follow-up data; however, no imputation methods or intention-to-treat analysis were performed. Furthermore, normality was assessed visually using histogram analysis, but additional formal normality testing, such as the Shapiro–Wilk test, was not conducted. As multiple chest compression outcomes were analysed without correction for multiple comparisons, the risk of type I error cannot be excluded, particularly for secondary outcomes.

Recruitment through convenience sampling in two Belgian companies may have further introduced selection bias and limited external validity. Finally, the use of a simulated manikin-based environment may not fully reflect real-world resuscitation conditions, potentially limiting the generalisability of the observed effects. This study attempted to minimise these biases through randomisation, standardised training procedures, objective automated outcome measurements and the use of predefined outcome measures.

Although real-time feedback devices may enhance selected CPR skills, their implementation in community training programmes should also be evaluated from an educational and economical perspective. The acquisition of feedback devices, instructor familiarisation and equipment maintenance may increase training costs compared with standard BLS courses.

Nevertheless, the objective automated recording of CPR parameters, standardised training protocol and predefined outcome measures strengthen the internal validity of the findings.

## 5. Conclusions

In adult laypersons, integrating a real-time feedback device into standard BLS training significantly improved compression rate and hand position at the 4-month follow-up compared with standard BLS training alone. No clinically meaningful results were observed for compression depth. Although overall CPR skill retention declined over time in both groups, using a real-time feedback device mitigated the skill decay in compression rate at follow-up. While these devices enhanced certain aspects of CPR quality and skill retention, they did not fully address all components of high-quality chest compressions and whether these improvements ultimately translate into improved survival and neurological outcomes following OHCA remains to be established. Combining feedback-assisted training with regular refresher sessions may strengthen bystander CPR performance and improve early resuscitation in OHCA. Future research should definitely focus on strategies to improve compression depth by exploring whether targeted physical conditioning, alternative feedback modalities, or extended training duration can address this gap. Furthermore, future research should determine optimal retraining intervals for lay rescuers as well as investigate the cost-effectiveness and feasibility of implementing feedback-assisted CPR training on a larger scale.

## Figures and Tables

**Table 1:** Demographic characteristics.

Characteristics	Control (*n* = 33)	Intervention (*n* = 68)	*p* Value
Sex			0.795
Male	7	16	
Female	26	52	
Age			0.150
18–25 y	0	3	
26–35 y	12	22	
36–45 y	7	25	
45–55 y	9	15	
55–65 y	5	3	
Previous BLS/ALS training (≥5 y ago)			0.420
Yes	9	24	
No	24	44	

BLS: Basic Life Support. ALS: Advanced Life Support.

**Table 2:** Within-group differences in chest compression parameters in the control group.

Chest Compression Parameters	Session 1 (Mean ± SD)	Session 2 (Mean ± SD)	*p* Value
Total number of compressions ^a^	275.36 ± 29.88	253.88 ± 42.83	<0.001 *
Mean compression rate ^b^ (min^−1^)	108.48 ± 12.45	94.03 ± 15.38	<0.001 *
Adequate compression rate ^c^ (%)	66.88 ± 34.34	35.52 ± 36.60	<0.001 *
Mean compression depth (cm)	5.06 ± 0.92	4.86 ± 0.98	0.315
Adequate compression depth ^d^ (%)	46.55 ± 33.80	39.94 ± 28.39	0.298
Correct hand position (%)	76.09 ± 34.39	73.64 ± 35.75	0.707
Full chest recoil ^e^ (%)	65.52 ± 30.92	78.36 ± 31.27	0.044 *
Flow fraction ^f^ (%)	64.88 ± 5.97	68.12 ± 8.58	0.046 *

^a^ Total number of compressions during 4-min test. ^b^ Mean compression rate is defined as the mean number of compressions per minute. ^c^ Adequate compression rate is the percentage of compressions within the optimal range of 100–120 compressions per minute. ^d^ Adequate compression depth is the percentage of compressions performed at 5–6 cm depth. ^e^ Full chest recoil is the percentage of compressions fully released. ^f^ Flow fraction is the percentage of time when chest compressions are performed without a pause ≥3 s. * Statistical significant.

**Table 3:** Within-group differences in chest compression parameters in the intervention group.

Chest Compression Parameters	Session 1 (Mean ± SD)	Session 2 (Mean ± SD)	*p* Value
Total number of compressions ^a^	295.65 ± 27.52	277.60 ± 43.26	0.001 *
Mean compression rate ^b^ (min^−1^)	113.07 ± 8.15	102.87 ± 15.37	<0.001 *
Adequate compression rate ^c^ (%)	72.88 ± 24.73	53.68 ± 39.05	<0.001 *
Mean compression depth (cm)	5.18 ± 0.59	5.06 ± 0.80	0.172
Adequate compression depth ^d^ (%)	54.62 ± 29.87	47.26 ± 30.36	0.040 *
Correct hand position (%)	93.31 ± 20.30	88.35 ± 29.59	0.257
Full chest recoil ^e^ (%)	54.50 ± 30.23	69.91 ± 32.14	<0.001 *
Flow fraction ^f^ (%)	66.35 ± 4.73	67.79 ± 5.79	0.055

^a^ Total number of compressions during 4-min test. ^b^ Mean compression rate is the mean number of compressions per minute. ^c^ Adequate compression rate is the percentage of compressions within the optimal range of 100–120 compressions per minute. ^d^ Adequate compression depth is the percentage of compressions performed at 5–6 cm depth. ^e^ Full chest recoil is the percentage of compressions fully released. ^f^ Flow fraction is the percentage of time when chest compressions are performed without a pause ≥3 s. * Statistical significant.

**Table 4:** Between-group differences of chest compression parameters in session 1.

Chest Compression Parameters	Control (Mean ± SD)	Intervention (Mean ± SD)	*p* Value
Total number of compressions ^a^	275.36 ± 29.88	295.65 ± 27.52	0.001 *
Mean compression rate ^b^ (min^−1^)	108.48 ± 12.45	113.07 ± 8.15	0.060
Adequate compression rate ^c^ (%)	66.88 ± 34.34	72.88 ± 24.73	0.374
Mean compression depth (cm)	5.06 ± 0.92	5.18 ± 0.59	0.474
Adequate compression depth ^d^ (%)	46.55 ± 33.80	54.62 ± 29.87	0.225
Correct hand position (%)	76.09 ± 34.39	93.31 ± 20.30	0.011 *
Full chest recoil ^e^ (%)	65.52 ± 30.92	54.50 ± 30.23	0.091
Flow fraction ^f^ (%)	64.88 ± 5.97	66.35 ± 4.73	0.182

^a^ Total number of compressions during 4-min test. ^b^ Mean compression rate is the mean number of compressions per minute. ^c^ Adequate compression rate is the percentage of compressions within the optimal range of 100–120 compressions per minute. ^d^ Adequate compression depth is the percentage of compressions performed at 5–6 cm depth. ^e^ Full chest recoil is the percentage of compressions fully released. ^f^ Flow fraction is the percentage of time when chest compressions are performed without a pause ≥3 s. * Statistical significant.

**Table 5:** Between-group differences and effect sizes in chest compression parameters in session 2.

Chest Compression Parameters	Control (Mean ± SD)	Intervention (Mean ± SD)	*p* Value	Cohen’s d ^g^
Total number of compressions ^a^	253.88 ± 42.83	277.60 ± 43.26	0.011 *	0.55
Mean compression rate ^b^ (min^−1^)	94.03 ± 15.38	102.87 ± 15.37	0.008 *	0.58
Adequate compression rate ^c^ (%)	35.52 ± 36.60	53.68 ± 39.05	0.028 *	0.47
Mean compression depth (cm)	4.86 ± 0.98	5.06 ± 0.80	0.267	0.23
Adequate compression depth ^d^ (%)	39.94 ± 28.39	47.26 ± 30.36	0.248	0.25
Correct hand position (%)	73.64 ± 35.75	88.35 ± 29.59	0.045 *	0.46
Full chest recoil ^e^ (%)	78.36 ± 31.27	69.91 ± 32.14	0.214	−0.27
Flow fraction ^f^ (%)	68.12 ± 8.58	67.79 ± 5.79	0.822	−0.05

^a^ Total number of compressions during 4-min test. ^b^ Mean compression rate is the mean number of compressions per minute. ^c^ Adequate compression rate is the percentage of compressions within the optimal range of 100–120 compressions per minute. ^d^ Adequate compression depth is the percentage of compressions performed at 5–6 cm depth. ^e^ Full chest recoil is the percentage of compressions fully released. ^f^ Flow fraction is the percentage of time when chest compressions are performed without a pause ≥3 s. ^g^ Cohen’s d values of 0.2, 0.5 and 0.8 represent small, medium and large effect sizes, respectively. * Statistical significant.

**Table 6:** Percentage of skill decay between sessions 1 and 2.

Chest Compression Parameters	Control (%)	Intervention (%)	Difference in Decay
Total number of compressions ^a^	−7.8%	−6.1%	+1.7%
Mean compression rate ^b^ (min^−1^)	−13.3%	−9.0%	+4.3%
Adequate compression rate ^c^ (%)	−46.9%	−26.3%	+20.6%
Mean compression depth (cm)	−4.0%	−2.3%	+1.7%
Adequate compression depth ^d^ (%)	−14.2%	−13.5%	+0.7%
Correct hand position (%)	−3.2%	−5.3%	−2.1%
Full chest recoil ^e^ (%)	+19.6%	+28.3%	+8.7%
Flow fraction ^f^ (%)	+5.0%	+2.2%	−2.8%

Percentage change calculated as ([S2 − S1]/S1) × 100. Positive ‘Difference in Decay’ indicates better retention in the intervention group. ^a^ Total number of compressions during 4-min test. ^b^ Mean compression rate is the mean number of compressions per minute. ^c^ Adequate compression rate is the percentage of compressions within the optimal range of 100–120 compressions per minute. ^d^ Adequate compression depth is the percentage of compressions performed at 5–6 cm depth. ^e^ Full chest recoil is the percentage of compressions fully released. ^f^ Flow fraction is the percentage of time when chest compressions are performed without a pause ≥3 s.

## Data Availability

The datasets generated and/or analysed during the current study are available from the corresponding author on reasonable request.
